# Autophagy in DNA Damage Response

**DOI:** 10.3390/ijms16022641

**Published:** 2015-01-23

**Authors:** Piotr Czarny, Elzbieta Pawlowska, Jolanta Bialkowska-Warzecha, Kai Kaarniranta, Janusz Blasiak

**Affiliations:** 1 Department of Molecular Genetics, University of Lodz, Pomorska 141/143, 90-236 Lodz, Poland; E-Mail: pczarny@biol.uni.lodz.pl; 2 Department of Orthodontics, Medical University of Lodz, Pomorska 251, 92-216 Lodz, Poland; E-Mail: elzbieta.pawlowska@umed.lodz.pl; 3 Department of Infectious and Liver Diseases, Medical University of Lodz, Kniaziewicza 1/5, 92-347 Lodz, Poland; E-Mail: jbialkowska@pro.onet.pl; 4 Department of Ophthalmology, Institute of Clinical Medicine, University of Eastern Finland, Kuopio FI-70211, Finland; E-Mail: Kai.Kaarniranta@kuh.fi; 5 Department of Ophthalmology, Kuopio University Hospital, Kuopio FI-70211, Finland

**Keywords:** autophagy, DNA damage response, DNA repair, apoptosis, signal transduction, senescence, cancer therapy

## Abstract

DNA damage response (DDR) involves DNA repair, cell cycle regulation and apoptosis, but autophagy is also suggested to play a role in DDR. Autophagy can be activated in response to DNA-damaging agents, but the exact mechanism underlying this activation is not fully understood, although it is suggested that it involves the inhibition of mammalian target of rapamycin complex 1 (mTORC1). mTORC1 represses autophagy via phosphorylation of the ULK1/2–Atg13–FIP200 complex thus preventing maturation of pre-autophagosomal structures. When DNA damage occurs, it is recognized by some proteins or their complexes, such as poly(ADP)ribose polymerase 1 (PARP-1), Mre11–Rad50–Nbs1 (MRN) complex or FOXO3, which activate repressors of mTORC1. SQSTM1/p62 is one of the proteins whose levels are regulated via autophagic degradation. Inhibition of autophagy by knockout of FIP200 results in upregulation of SQSTM1/p62, enhanced DNA damage and less efficient damage repair. Mitophagy, one form of autophagy involved in the selective degradation of mitochondria, may also play role in DDR. It degrades abnormal mitochondria and can either repress or activate apoptosis, but the exact mechanism remains unknown. There is a need to clarify the role of autophagy in DDR, as this process may possess several important biomedical applications, involving also cancer therapy.

## 1. Introduction

**Figure 1:**
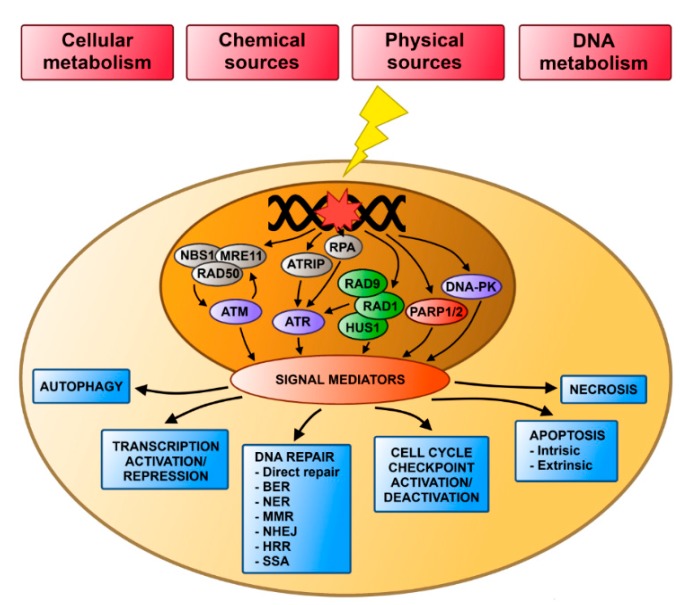
Cellular response to DNA damage. DNA damage can be induced by exogenous chemical and physical factors, or by endogenous influences following from cellular and DNA metabolism. The induction of DNA damage triggers the DNA damage response (DDR). Three proteins from the phosphatidylinositol 3-kinase-like protein kinases (PIKKs) family plays a major role in DDR: ataxia telangiectasia mutated (ATM), DNA protein kinase (DNA-PK) and ataxia telangiectasia and Rad3 related (ATR), two proteins of the poly(ADP-ribose) polymerase (PARP) family: PARP1 and PARP2, and heterotrimeric complex of Rad9, Rad1 and Hus1 (9–1–1 complex). These proteins are activated by either DNA damage itself or by other proteins. After ATM, ATR, DNA-PK, PARP1/2 or 9–1–1 complexes are activated, they transfer signals via signal mediators to regulate many cellular processes, including DNA repair, cell checkpoint activation or deactivation, activation or silencing of transcription, apoptosis and autophagy.

Cells respond to different stress stimuli in order to survive, duplicate and avoid cancer transformation. The DNA damage response (DDR) plays an important role against detrimental effects of stress. It coordinates many processes, including DNA repair, regulation of cell cycle checkpoints, transcription of DDR genes, and ultimately induction of a programmed cell death, most often apoptosis, when DNA damage cannot be repaired (Figure 1). A growing body of evidence suggests, that autophagy, a catabolic process considered to be a cellular survival mechanism, may also play a role in DDR.

## 2. DNA Damage and Its Cellular Response

### 2.1. DNA Damage and Repair

DNA damage can be induced by a variety of physical and chemical factors, generated exogenously or endogenously, by ultraviolet (UV) light and ionic radiation (IR) as well as metabolic reactions, producing reactive oxygen and nitrogen species (ROS and RNS, respectively) [1,2,3].

If a DNA damage is left unrepaired or is misrepaired, it can be changed into a mutation, which may play a role in pathogenesis of diseases, including cancer [3,4,5]. Therefore, an accurate DNA repair system is important for normal life of the cell. Some DNA damages can be repaired in a simple one step chemical reaction. This kind of repair pathway is known as DNA damage direct reversal. In this pathway methylated bases can be demethylated using the “suicide” enzyme *O*(6)-methylguanine-DNA methyltransferase (MGMT) [6,7]. Another two pathways, base excision repair (BER) and nucleotide excision repair (NER) can restore single-strand DNA damage. BER removes small chemical modifications, such as those caused by oxidative bases, while NER can repair damage affecting more than one DNA base, e.g., pyrimidine dimers (Figure 1). Mismatch repair (MMR) replaces a wrongly incorporated nucleotide with the correct one [8,9]. DNA double-strand breaks (DSBs), belonging to the most serious DNA damage, can be repaired by three pathways: homologous recombination repair (HRR), non-homologous end joining (NHEJ) and single-strand annealing (SSA), along with their several variants [10].

Mitochondrial DNA (mtDNA) is more prone to damage than its nuclear counterpart (nDNA) [11,12,13]. First, due to its close proximity to the electron transport chain (ETC), mtDNA is exposed to a relatively high level of ROS and RNS. Second, mtDNA lacks histones and non-histone proteins associated with DNA, forming a “molecular shield” protecting nDNA from damage. Third, mitochondrial mechanisms of DNA repair are less efficient and limited when compared to their nuclear analogues. Moreover, the repair of mtDNA is usually performed at sites of ROS generation. In addition, many reports suggest the lack of efficient NER in mitochondria [14]. Since mtDNA is especially prone to ROS- and RNS-induced lesions, BER is the main pathway activated in response to mtDNA damage [15,16]. Other pathways are also present in mitochondria, but their mechanisms are less known and may differ from their nuclear counterparts [17,18,19,20].

### 2.2. DNA Damage Signaling

Fast and precise transduction of the DNA damage signal is crucial for the efficiency of its repair. This signal is transduced mainly by a cascade of phosphorylation/dephosphorylation reactions [21].

Proteins from the phosphatidylinositol 3-kinase-like protein kinases (PIKKs) and poly(ADP)ribose polymerase (PARP) families play the major role in DDR signaling (Figure 1) [22,23]. ATM (Ataxia telangiectasia mutated) and DNA-PK (DNA-dependent protein kinase) are recruited to the site of DSBs [24,25]. Unlike DNA-PK, which only coordinates proteins responsible for DSB end joining, ATM controls more processes, including DNA replication, transcription, metabolic signaling and DNA splicing [1,26,27]. After creating a complex with ATR (Ataxia telangiectasia mutated and Rad3-related protein)-interacting protein (ATRIP) ATR recognizes persistent single-strand DNA (ssDNA) coated with replication protein A (RPA), which is present at stalled replication forks and DSBs [28]. Similarly to ATM, ATR regulates also other important cellular processes [26,27]. The 9–1–1 complex (heterotrimeric complex of Rad9, Rad1 and Hus1) and Rad17 are also involved in the detection of ssDNA coated with RPA. This complex creates a ring structure resembling the proliferating cell nuclear antigen (PCNA), and Rad17 shares a high homology with replication factor C (RFC) [29,30,31,32,33,34,35]. Rad17 recruits 9–1–1 to the DNA damage in a similar way that RFC engages PCNA. Two members of PARP (poly(ADP-ribose) polymerase) protein family involved in the DDR build chains of poly(ADP-ribose) (PAR) in the regionof single-strand breaks (SSBs) and DSBs occurrence to recruit other DDR proteins [36].

The involvement of ATM and ATR in DDR has been described (Figure 1). ATM, in its inactive form, creates a homodimer, which is recruited to the site of DSB by the Mre11–Rad50–Nbs1 (MRN) complex, then undergoes autophosphorylation and separates into two active monomers [37]. After recruitment and activation, ATM and ATR interact with many mediator and executing proteins, including Checkpoint kinase 1 and 2 (CHK1 and CHK2) involved in the cell cycle control, p53, a multifunctional protein essential for cell survival, breast cancer type 1 susceptibility protein (BRCA1)-associated genome surveillance complex (BASC) containing DNA damage repair proteins, histone deacetylases 1 and 2 (HDAC1 and HDAC2) responsible for remodeling the structure of chromatin, and transcription factor FOXO3, regulating genes involved in DNA repair [38,39,40,41,42].

### 2.3. Programmed Cell Death and DNA Damage

When DNA damage is left non-repaired, it may induce cell transformation or death. Morphologically, two different types of cell death: necrosis and programmed cell death, most often apoptosis, can be considered (Figure 1) [43]. Necrosis is characterized by an enlargement of cell volume (oncosis) and swelling of organelles. When oncosis reaches a critical point, the cell membrane breaks and the entire content of the cell flow into the extracellular space, often leading to inflammation. Although it is assumed that necrosis is an uncontrolled process occurring after overwhelming stress, there are some data suggesting that it can be regulated to some extent [44]. Apoptosis involves chromatin condensation, fragmentation of the nucleus, plasma membrane blebbing and creation of apoptotic bodies [45]. Triggering of apoptosis may occur by extrinsic or intrinsic pathways. Briefly, apoptosis via the extrinsic pathway is activated by death receptors belonging to the tumor necrosis factor receptor (TNFR) gene superfamily containing the evolutionary conserved death domain (DD). These receptors, such as TNFR-1/TNF-α or Fas/CD95, become activated when they bind specific ligands, form trimmers, and transduce signals via cytoplasmic death receptors. The signal from FAS receptors is transduced via Fas-Associated protein with death domain (FADD) and the signal from TNF receptors via TNFR-1-associated death domain protein (TRADD) with additional recruitment of FADD and receptor-interacting protein kinase (RIP) [46,47]. Similarly to DD, FADD contains a conserved motif called the Death Effector Domain (DED), which is also present in procaspase-8. Due to dimerization of these domains, FADD recruits procaspase-8 and this creates a death-inducing signaling complex (DISC). After DISC is formed, procaspase-8 undergoes auto-catalytic activation and, as caspase-8, triggers the implementation phase of apoptosis [48]. The intrinsic pathway is also called a mitochondrial pathway, because its crucial step is the release of pro-apoptotic proteins from the mitochondrial intermembrane space into the cytosol, (mitochondrial outer membrane permeabilization (MOMP) [49]. Thus, MOMP liberates the Smac/DIABLO (direct IAP (the inhibitor of apoptosis protein)-binding protein with low pl) complex, which promotes apoptosis by suppressing the inhibitors of apoptosis (IAP) and cytochrome *c*, which together with Apaf-1 and procaspase-9 creates the apoptosome, leading to caspase-9 activation [50]. There are also other pro-apoptotic proteins, including AIF (apoptosis-inducing factor), endonuclease G and CAD, which are responsible for DNA fragmentation [51]. The process of MOMP is regulated by members of the Bcl-2 family, containing pro- and anti-apoptotic proteins [52].

Severe DNA damage can induce both the extrinsic and intrinsic apoptosis pathways. As mentioned above, in response to DNA damage, ATM and ATR are activated and transduce signals via phosphorylation to other proteins, including p53. This chemical modification increases stability of p53, which can trigger apoptosis via mitochondria either as a transcription activator of pro-apoptotic proteins BAX (Bcl-2-assciated X protein 1), BID (BH3 interacting-domain death agonist), NOXA (Phorbol-12-myristate-13-acetate-induced protein), PUMA (p53 upregulated modulator of apoptosis) and FAS (tumor necrosis factor receptor superfamily 6) or by binding to anti-apoptotic proteins of the Bcl-2 family [53,54]. Moreover, ATR participates in phosphorylation of BRCA1 in response to UV light [55,56,57,58]. BRCA1, a protein involved in homologous recombination repair (HRR) and non-homologous end joining (NHEJ) DNA repair pathways, was found to stimulate apoptosis in a p53-independend manner [59,60]. DNA-PK can also induce apoptosis via phosphorylation of p53 [61]. This induction occurs in response to severe DNA damage or due to critically shortened telomeres [62,63]. Another protein involved in DDR and apoptosis is PARP1 [64]. It was shown that inhibition of PARP1 in combination with inhibition of epidermal growth factor receptor (EGFR) induces intrinsic apoptosis [65]. This approach has been recently translated pre-clinically as EGFR inhibition reduced HRR and NHEJ pathways and PARP1 inhibition thus augments the effect of chemotherapy as well as targeted radionuclide therapy [66]. In addition, PARP1 inhibition triggers caspase-independent cell death by mitochondrial release of AIF, which causes nDNA fragmentation [67,68].

It should be noted that programmed cell death in animals most often, but not exclusively, occurs by apoptosis and several other programmed pathways can be considered, including autophagy, anoikis excitotoxicity, ferroptosis, cornification and Wallerian degeneration. Therefore, there are many effector pathways exist downstream of DDR, including autophagy. However, autophagy can also contribute to survival mechanism of the cell after genotoxic insult, along with other mechanisms, including cell cycle arrest and mitotic arrest as well as reversible senescence (see [69] for review).

## 3. Autophagy

Autophagy, from Greek literally meaning “self-eating”, is a tightly regulated, evolutionary conserved, catabolic process, in which damaged proteins and organelles are degraded in lysosomes [70]. Three basic types of autophagy can be considered: microautophagy, chaperon-mediated autophagy (CMA) and macroautophagy [69,71,72]. Microautophagy is a nonselective process sequestering cytosolic proteins via invagination of the lysosomal membrane [73,74]. In CMA, proteins are selectively delivered to the lysosomes after recognition of their consensus sequence by a molecular chaperones, for example by HCS70 (heta-shock cognate) [75,76]. Macroautophagy which will be subsequently referred to as “autophagy” starts with the formation of autophagosome, a double-membrane vacuole, which encloses bulk proteins and organelles in the cytosol and delivers them to lysosome for degradation, resulting in the release of amino acids and fatty acids that can be reused by the cell [77,78,79]. Autophagy is triggered in response to the various stress stimuli, including nutrient and energy stresses, endoplasmic reticulum (ER) stress, hormone stimulation, hypoxia, redox stress, mitochondrial damage and DNA damage [80]. Although autophagy is considered to be a major protective mechanism against stress stimuli and plays an important role in many physiological processes, extensive autophagy may lead to cell death [43,81]. Autophagic cell death is characterized by the presence of many autophagic structures and, in the contrast to apoptosis, chromatin condensation occurs in the later steps, there is no DNA fragmentation or formation of apoptotic bodies and debris of cell are phagocytized later or there may be no remnants to be phagocytized at all [81].

One of the best described pathways leading to autophagy is activated during starvation. In this pathway mammalian target of rapamycin (mTOR) plays a central role as a negative regulator of autophagy. mTOR complex 1 (mTORC1) is associated with another complex of proteins consisting of ULK1 (Unc-51-like kinase 1), which is a mammalian analog of the yeast master regulator of autophagy, Atg1, the mammalian analog of Atg13 and the scaffold protein FIP200 (FAK (focal adhesion kinase)-family interacting protein of 200 kDa) [81,82]. Inhibition of autophagy is achieved by the phosphorylation of ULK1 and Atg13 by mTOR [83,84]. During starvation, mTORC1 dissociates from the Atg13–FIP200–ULK1 complex leading to dephosphorylation of ULK1 and Atg13, activation of ULK1, which phosphorylates Atg13 and FIP200 [85,86,87]. After activation of autophagy, a structure called the pre-autophagosomal membrane, which later will become the autophagosome, is formed. The pre-autophagosome is created by the complex of Atg5–Atg12–Atg16L. Atg5 is an E3 ubiquitin ligase, which is conjugated to the Atg12 protein [88]. The conjugate interacts with Atg16L to form a multimeric complex by homo-oligomerization of Atg16 [89]. The process of autophagosome maturation is controlled by the class III PI3 kinase (PI3KIII) Vps34, which forms complex with Beclin-1 and Vps15/p150, and its kinase activity is enhanced by binding with UVRAG (UV radiation resistance-associated gene) and Bif-1 (BAX-interacting factor-1) [90,91,92,93,94]. Vps34 interacts with Atg14 and targets it to the pre-autophagosome membrane, where Atg14 recruits Atg16 and LC3 [95]. LC3 is the mammalian analog of Atg8 and its cytoplasmic form, LC3-I is lipidated upon conjugation with phosphatidyl-ethanolamine (PE) to form LC3-II, which is responsible for expanding the autophagosome membrane [94,95]. This process is promoted by the Atg5–Atg12–Atg16L complex. After the autophagosome is assembled, it merges with the lysosome, where proteins and organelles are degraded into amino acids and fatty acids. The process of fusion is regulated by the pH of organelles’ interior: low pH promotes fusion, whereas an alkaline pH inhibits this process [95].

## 4. DNA Damage Response and Autophagy

An emerging body of evidence suggest that autophagy can play an important role in DDR (Figure 1). It was observed that after exposure to DNA-damaging agents, including ionizing radiation (IR), etoposide, camptothecin, tomozolomide or *p*-anilioaniline, not only the cell cycle was stopped, but also autophagy was induced [96,97,98]. Autophagy plays a cytoprotective role during anticancer therapy with DNA-damaging agents and its inhibition can sensitize cancer cells to these agents [66,99,100,101,102,103]. Additionally, the suppression of autophagy results in chromosomal instability, especially after metabolic stress, which may result in oncogene activation and tumor progression [102,103,104].

### 4.1. Autophagy and DNA Damage Response in the Nucleus

**Figure 2:**
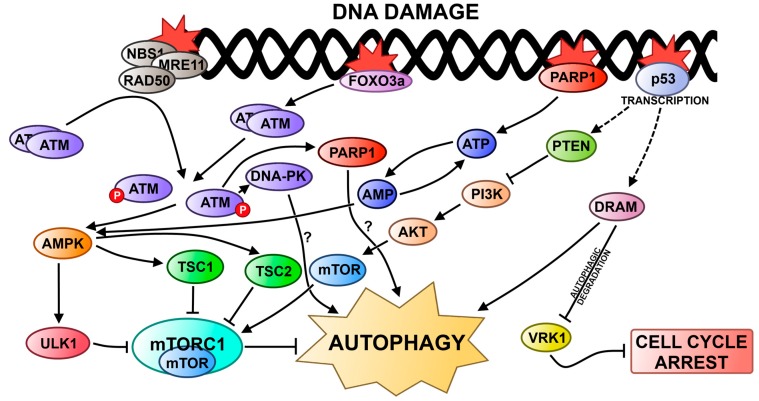
Autophagy in the DNA damage response. Activation of autophagy in response to DNA damage is mainly achieved by inhibition of the mTOR complex **1** (mTORC1), which is a negative regulator of autophagy. p53 acts as a transcription factor, and if there is DNA damage it can upregulate expression of proteins inducing autophagy. One of them, DRAM (damage-regulated autophagy modulator), is responsible for autophagy degradation of VRK1 (vaccina-related kinase 1), a protein involved in cell cycle checkpoint activation, thus it arrests the cell cycle.

ATM is activated in response to DNA damage by the MRN complex and it plays a key role in DDR (Figure 1 and Figure 2). Activation of ATM after exposure to genotoxic and oxidative agents causes repression of mTORC1 and induction of autophagy [105,106]. The transduction of the signal from ATM to mTORC1 is mediated via the AMPK (5' adenosine-monophosphate-activated protein kinase) pathway, which involves tuberous sclerosis complex 1 and 2 (TSC1/2) and phosphorylation of ULK1 [107,108,109,110]. ATM can be also activated by the transcriptional factor FOXO3a (forkhead box O3) via phosphorylation [111]. Under normal conditions, FOXO3a is attached to DNA, but upon DNA damage, it dissociates from DNA and interacts with ATM promoting DNA repair. Additionally, FOXO3a regulates transcription of autophagy-related genes, including LC3 or Bnip3 [112,113,114].

The p53 protein is crucial for DDR and plays an important role in the regulation of autophagy [115,116,117,118,119,120,121,122,123,124].

PARP-1 is another protein of DDR involved in the regulation of autophagy. As described above, after a DNA lesion, PARP-1 synthesizes poly(ADP-ribose) chains that recruit the DNA damage repair proteins (Figure 2). On the other hand, when PARP-1 is hyperactivated in response to DNA damage, it causes NAD^+^ depletion leading to cellular energy failure and necrotic cell death [125,126]. Recently, it was shown that during starvation conditions, ROS-induced DNA damage activated PARP-1, which caused a depletion of ATP and activation of AMP-activated protein kinase (AMPK) [127]. Then AMPK sensed the energy depletion by measuring the ratio of AMP to ATP and it inhibited mTOR via TSC1/2, thus inducing autophagy [128]. In addition, H_2_O_2_- and doxorubicin-induced DNA damage activates a pathway involving PARP-1, which can induce either necrosis or autophagy [129,130]. Similarly, PARP-1 together with the catalytic subunit of DNA-PK (DNA-PKcs) was found to be activated by ATM during the autophagy induced by a chemopreventive agent, capsaicin, leading to DNA repair and the survival of breast cancer MCF-7 cells [130].

Results of recent research suggest that sirtuins, a family of protein deacetylases dependent on NAD^+^, may play an important role in autophagy and DDR [131]. The mammalian sirtuins family consists of 7 members, SIRT1–7, but in the light of recent studies, SIRT1 seems to play the most important role in autophagy/DDR, first of all due to its involvement in cellular reaction to oxidative stress and programmed death [132]. SIRT1 can induce the formation of autophagosome by interaction and deacetylation of the Atg5, Atg7 and Atg8 proteins in a NAD^+^-dependent fashion [133]. In addition, mediators of autophagy, mTOR1 and FOXO may be targeted by SIRT1 [134,135]. SIRT1 interacts with many protein which can be, directly or indirectly, involved in DDR, but its interaction with p53 seems to be crucial for its role in DDR, because it affects transcriptional activity of p53 regulating expression of p53 downstream effectors important for cell cycle regulation and programmed death under DNA-damaging conditions [136]. Therefore, SIRT1 may be involved in the regulation of autophagy in nontoxic stress, but precise mechanism underlying this involvement is not known and requires further studies.

**Figure 3:**
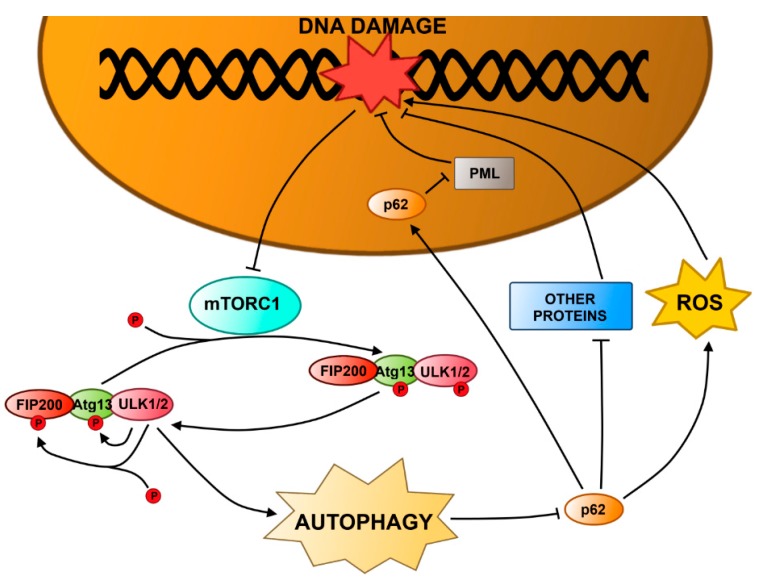
FIP200 (FAK (focal adhesion kinase)-family interacting protein of 200 kDa) and p62 in autophagy and DNA damage response. The mTOR1 (mammalian target of rapamycin) complex (mTORC1) interacts with ULK1/2 (UNC-51-like kinase 1/2)–Atg13 autophagy-related protein 13)–FIP200 complex and phosphorylates Atg13 and ULK1/2. If there is DNA damage, mTORC1 is inhibited, causing a slow decrease in phosphorylation of Atg13 and ULK1/2. Unphosphorylated ULK1/2 exhibits its kinase activity triggering phosphorylation of FIP200 and Atg13 and activating the ULK1/2–Atg13–FIP200 complex. The complex translocates to the pre-autophagosomal structure promoting autophagy. p62, a multifunctional ubiquitin-binding protein, is degraded by autosphagy. Inhibition of autophagy up-regulates p62, which causes an increase in the amount of DNA damage. The increase is caused either by generation of reactive oxygen species (ROS) or inhibition DNA repair via a direct interaction of p62 with promyelocytic leukemia (PML) nuclear bodies containing DDR proteins or an indirect interaction with other proteins in the cytosol.

FIP200, a 200 kDa FAK-family interacting protein, is a multifunctional protein regulating many cellular processes, including proliferation, cell migration and apoptosis, by interacting with FAK, Pyk2 (proline-rich tyrosine kinase 2), TSC1, p53, ASK1 (apoptosis signal-regulating kinase 1) or TRAF2 (TNF receptor-associated factor 2) [137,138,139,140] In addition, FIP200 is a component of the ULK1/2–Atg13–FIP200 complex and is essential for activation of autophagy (Figure 3). This complex is directly regulated by mTORC1 [141,142,143]. Under normal conditions, mTORC1 interacts with the complex by phosphorylation of ULK1/2 and Atg13, but when mTORC1 is inhibited, the level of phosphorylation of ULK1/2 and Atg13 decreases. This results in an increased kinase activity of ULK1/2, subsequent phosphorylation of Atg13 and FIP200, and translocation of the ULK1/2–Atg13–FIP200 complex to pre-autophagosomal structures [144,145]. Recently, it was shown that mouse embryonic fibroblasts (MEFs) with FIP200 knockout (KO) displayed a less efficient repair of the DNA damage induced by IR and two anticancer agents, camptothecin and etoposide, compared to the wild-type cells [146]. Moreover, KO of FIP200 caused up-regulation of SQSTM1 (sequestome 1)/p62 expression and formation of aggregates containing SQSTM1/p62. Re-expression of FIP200 restored the wild phenotype in FIP200 KO MEFs and suppressed SQSTM1/p62 expression. This indicates, that FIP200 regulates DNA damage response by autophagy and regulation of SQSTM1/p62 expression [147,148,149].

SQSTM1/p62 is an ubiquitin binding and scaffolding protein [141]. Its multiple domain structure enables controlling many processes, including osteoclastogenesis, inflammation, differentiation, neurotrophin properties and obesity [150]. SQSTM1/p62 is also involved in selective degradation via autophagy [151]. Damaged or unfolded proteins are polyubiquitinated, which recruits SQSTM1/p62 and induce binding to Atg8/LC3 presented on autophagosome membrane that finally leads to autophagic degradation of the aggregates [151]. It is not known how SQSTM1/p62 regulates the efficiency of DDR. SQSTM1/p62 is localized mainly in the cytoplasm, but it has a nuclear export signal (Figure 3). In the nucleus, it interacts with promyelocytic leukemia (PML) nuclear bodies that contain DDR proteins: BLM (Bloom syndrome)/WRN (Werner syndrome) DNA helicases, MRN or TopBP1 (DNA topoisomerase II binding protein), and are involved in DDR [152,153]. On the other hand, no significant relocalization of SQSTM1/p62 was observed and its influence on DNA repair efficiency may be indirectly mediated by an interaction with other proteins in the cytoplasm via its scaffold function. Additionally, up-regulation of SQSTM1/p62 expression causes increased ROS production, which may also contribute to increased DNA damage and create an amplification loop [154].

### 4.2. Autophagy and DNA Damage Response in Mitochondria

**Figure 4:**
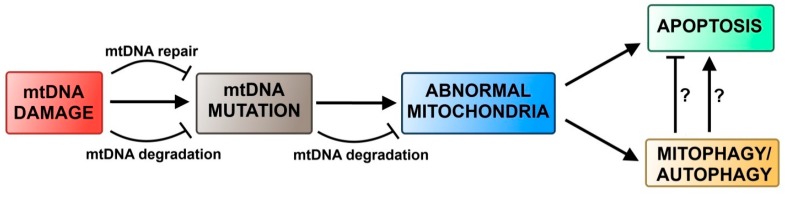
Autophagy in mitochondrial DNA damage response. Damaged mitochondrial DNA (mtDNA) can be either repaired or degraded. This prevents transition of the damage to a mutation. When a mutation occurs, it can cause degradation of mutated mtDNA. Nevertheless, replication of mutated mtDNA molecules can cause a decrease in the mutation threshold and result in abnormalities in mitochondrial physiology. On the one hand, such abnormalities can cause apoptosis via the intrinsic pathway but on the other hand, these damaged mitochondria can be degraded via mitophagy. Question marks denote hypothetical pathways.

As mentioned above, DNA repair systems in mitochondria are less efficient, when compared to their nuclear counterparts. Although the mitochondrial genome contains only 37 genes, mutations and deletions of mtDNA are responsible for a significant number of inherited mitochondrial diseases, indicating the importance of mtDNA integrity for human health [155,156]. Damaged mitochondria may produce elevated levels of ROS, thus inducing even more DNA damage [43]. Since there are more than one mitochondrion in a cell and each mitochondrion has several copies of mtDNA, the damaged molecules can be degraded in live cells. It has been shown that degradation of mitochondria and mtDNA can be executed by a selective autophagic pathway, called mitophagy (Figure 4). The receptor to ensure the selectivity of mitophagy is the Nix protein [157]. After recruitment of Nix in response to mitochondrial depolarization, these mitochondria are marked for degradation by ubiquitination of this mitochondrial protein by the E3 ligase Parkin [158,159,160]. Recently, it was shown that SQSTM1/p62 binds to ubiquitinated mitochondrial membrane through its ubiquitin-binding domain and recruits the pre-autophagosome by LC3 binding domain [161,162,163,164]. The importance of SQSTM1/p62 in mitophagy remains to be elucidated. On the one hand, knockout of SQSTM1/p62 disabled elimination of mitochondria with compromised membrane potential, but other studies showed that SQSTM1/p62 is only involved in mitochondria aggregation, not in mitophagy itself. Nevertheless, it has been reported that the inductions of autophagy and mitophagy were triggered by toxic exposure, mtDNA mutations, ROS and UV [165,166,167]. In addition, blockage of autophagy and mitophagy can result in the accumulation of dysfunctional mitochondria, damaged mtDNA and an increased rate of apoptotic cell death [168,169,170]. In yeast mutations causing mitochondrial dysfunctions, especially these impairing the mitochondrial electrochemical transmembrane potential, have induced mitophagy even during nonstarvation conditions [171]. A similar observation was made in mammalian cells, when either mutations in mtDNA or drug-induced loss of mitochondrial membrane potential caused mitochondrial elimination by autophagy [172,173]. Autophagy may play a protective role against apoptosis, because it eliminates damaged mitochondria, thus preventing them from releasing proapoptotic proteins [173]. On the other hand, in the presence of caspase inhibitors, elimination of mitochondria by autophagy is crucial in triggering cell death [174]. Overall, these findings indicate that mtDNA damage can trigger mitophagy in an indirect way by the induction of mutations and changes in mitochondrial physiology, rather than by any direct signal.

## 5. Conclusions and Perspectives

Autophagy is a central player in the regulation of DDR. Impairments in this process have been connected to increased susceptibility of the cells to genotoxic agents, which may be important in anticancer therapy. It was shown that a DNA topisomerase II and tyrosine kinase 3 inhibitor induced autophagy in cancer cell lines and this process was associated with acquiring of senescent phenotype, which might be essential for a cytostatic action of this drug [175]. Senscence, associated with stable cell arrest, does not inhibit cellular apoptosis, as in apoptosis [176]. Genotoxic stress, leading to activation of DDR, may evoke autophagy as an early adaptative response, which can be compared with the DDR mechanisms of DNA damage tolerance, but this issue needs further research and explanation. Regulation of mechanisms of cross-talk between autophagy and apoptosis and senescence may be important for the regulation of DDR and cell fate and should be further studied as some controversial results were obtained so far. As mentioned, autophagy in DDR may be determined by the involvement of p53, a multifunction tumor suppressor, which is essential for determining the cell fate after a stress stimulus, but which mechanism of anticancer action is not fully known. Recent studies revealed several novel elements of p53 and a large autophagy network regulated by p53 and its family members, first of all p63 and p73 [176]. These studies revealed that when activated by p53, autophagy did not promote survival, but induced p53-dependent apoptosis. Therefore, further studies on the role of p53 in autophagy in the context of DDR may bring some important information on tumor suppressor role of p53 with potential relevance to anticancer therapy. Although many lines of evidence suggest the feasibility of autophagy as a target in anticancer therapy, it should be taken into account that this process may play a context-dependent role in cancer development. On one hand, its involvement in DDR may induce apoptosis and prevent genomic instability, which is a hallmark of cancer transformation, but on the other hand, it may promote survival of cancer cells in unfavorable, stress conditions, including those following from anticancer therapy. Autophagy is seen as a pro-survival mechanism due to its critical role in maintaining cellular protostasis and in the regulation of inflammation and cell death in conditions of metabolic stress in tumor cells [177]. In contrast, inhibition of autophagy has been shown to sensitize tumor cells to the cytotoxic effects of both chemotherapy and irradiation and thus it can improve the results of these kinds of cancer treatment [178]. Autophagy cell death is one of the standard cell death mechanisms. Therefore, a powerful promotion of autophagy by drugs would be predicted to achieve a better therapeutic efficacy. Induction of autophagic cell death might be a therapeutic aim if the apoptotic signaling is defective in tumor cells. Thus, the dual role of autophagy in tumor cells is not only of clinical interest but it also provides opportunities for the development of novel chemotherapeutic strategies. Various autophagy-regulating drugs could function as many different ways e.g., photosensitizors, lysosomotrophic agents, apoptosis inducers, proton pump inhibitors, toll-like receptor agonists, microtubule depolymerizators, cell cycle controllers, ROS generators, mTOR kinase inhibitors, tyrosine kinase inhibitors, AMP-kinase regulators and histamine receptor antagonists; all of these kinds of agents have been evaluated a potential cancer therapy alternatives [178,179,180]. However, the question about exact role of autophagy in DDR and its implications for cancer therapy is still waiting for the answer.
